# Imaging Nanoscale Electromagnetic Near-Field Distributions Using Optical Forces

**DOI:** 10.1038/srep10610

**Published:** 2015-06-15

**Authors:** Fei Huang, Venkata Ananth Tamma, Zahra Mardy, Jonathan Burdett, H. Kumar Wickramasinghe

**Affiliations:** 1 Department of Electrical Engineering and Computer Science, University of California, Irvine, CA 92697, USA; 2 CaSTL Center, Department of Chemistry, University of California, Irvine, USA

## Abstract

We demonstrate the application of Atomic Force Microscopy (AFM) for mapping optical near-fields with nanometer resolution, limited only by the AFM probe geometry. By detecting the optical force between a gold coated AFM probe and its image dipole on a glass substrate, we profile the electric field distributions of tightly focused laser beams with different polarizations. The experimentally recorded focal force maps agree well with theoretical predictions based on a dipole-dipole interaction model. We experimentally estimate the aspect ratio of the apex of gold coated AFM probe using only optical forces. We also show that the optical force between a sharp gold coated AFM probe and a spherical gold nanoparticle of radius 15 nm, is indicative of the electric field distribution between the two interacting particles. Photo Induced Force Microscopy (PIFM) allows for background free, thermal noise limited mechanical imaging of optical phenomenon over wide range of wavelengths from Visible to RF with detection sensitivity limited only by AFM performance.

Atomic Force Microscopy (AFM)^1^, enables high resolution imaging of the physical^1^, chemical^2^, magnetic^3^, and electrostatic^4^ properties of materials at the nanoscale. Previously, a novel AFM based Photo Induced Force Microscopy (PIFM) scheme was introduced to detect and image molecular resonances at nanometer level^5^,^6^,^7^,^8^ and produce nanoscale optical force maps^9^,^10^. Near-field Scanning Optical Microscopy (NSOM) has been used to study light-matter interaction beyond the diffraction limit with important applications in many areas of nano-optics, materials science, chemistry and biology^11^. Both aperture NSOM (*a*-NSOM)^12^ and aperture-less or scattering NSOM (*s*-NSOM)^13^ techniques have been used to map the nanoscale electric^14^,^15^,^16^,^17^ and magnetic^18^,^19^,^20^ field distributions. s-NSOM has also been used to map the plasmonic modes in nanostructures and nanoantennas^21^,^22^,^23^,^24^. NSOM techniques involve the sampling of evanescent electromagnetic fields close to the sample surface due to light scattering by structured metallic probes brought close to the surface of the sample. Scattered evanescent fields are converted into propagating modes that are detected in the far-field. While many techniques have been proposed for reducing background noise, fundamentally, NSOM techniques are limited by the sensitivity of the schemes used for far-field light collection and detection. In contrast, PIFM can detect and image nanoscale light-matter interactions without the need for any far-field light collection. PIFM detection is fundamentally wavelength independent and can be applied to measure both linear^5^,^7^ and non-linear responses^6^ of materials. It extends the domain of AFM to include optically induced forces with applications in nano/atomic scale optical imaging and microscopy. In this paper, we use the tapping mode AFM working under ambient conditions to investigate and map the nanoscale electromagnetic field distributions with resolution limited only by the AFM probe geometry. By detecting the optical force between a gold coated AFM probe illuminated by a tightly focused laser beam and its image induced on a clean glass substrate, we are able to map the electric field distributions within the focal region. The experimentally obtained focal force distribution is in agreement with theoretical predictions from a simple dipole-dipole interaction model. Using only optical forces and by fitting to a dipole-dipole model, we experimentally estimate the aspect ratio of the apex of a gold coated AFM probe (in terms of the ratio of polarizabilities of the tip apex) modeled as a prolate spheroid. In addition, we map the interaction forces between a gold sphere of radius 15 nm and a gold coated AFM probe illuminated by an incident electric field. The measured force distributions are in excellent agreement with numerical calculations validating our experimental results. The experimental results are found to be background free, limited only by the thermal noise and AFM performance. Optical forces due to both electric and magnetic dipole-dipole interactions can be measured using this technique.

The origin of optical forces in PIFM can be well understood by modeling the sample under measurement as a sub-wavelength magneto-dielectric nanoparticle (modeled as a prolate spheroid with electric and magnetic dipole moments
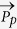
and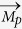, respectively) and the tip of the AFM probe as a sub-wavelength magneto-dielectric nanoparticle (modeled as a prolate spheroid with electric and magnetic dipole moments 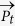 and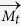, respectively) . We assume electromagnetic fields (with electric field 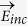 and magnetic field
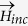
incident along the *z* direction) are tightly focused at the interface between the background medium (with isotropic relative permittivity and permeability 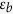 and 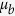, respectively) and the substrate (with isotropic relative permittivity and permeability 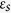
and 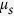, respectively) as shown schematically in Fig. 1 (a).

The total force experienced by the AFM probe tip is
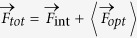
 , where, 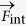 is the total tip-sample interaction forces consisting of all van der Waals forces, meniscus forces, chemical and Casimir forces, and 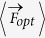 is the time-averaged optical force on the AFM probe tip due to its interaction with the incident field and particle dipole. 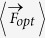 due to the presence of both electric and magnetic dipoles, can be written as the sum, 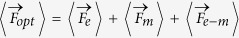
25,26 ,where, 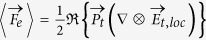 is the time-averaged force experienced due to the electric dipole with assumption of spatially non-varying moment 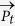, 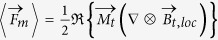 is the time-averaged force experienced due to magnetic dipole with assumption of spatially non-varying moment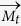, 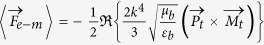 is the interaction force due to coupling between the electric and magnetic dipoles and ***k*** is the propagation constant of the incident wave. Here, 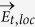 and 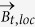 are the local electric and magnetic fields experienced by the AFM probe tip respectively and are given by the sum of the incident field and the fields scattered by the particle and the substrate.

Proper choice of 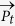
and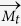 could allow for the detection of optical electric and/or magnetic fields using forces. For example, the use of a metal coated tip supporting large values of 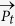 could be used to measure the electric dipole-dipole interaction forces schematically shown in Fig. 1 (b). Due to the weak nature of magnetism in materials at optical frequencies, magnetic dipole-dipole interaction forces could be detected by measuring the interaction forces between nanostructures supporting artificial magnetism. In particular, it is well known that nano-spheres composed of dense dielectric materials support the magnetic Mie resonance^27^. In addition, metal nanoparticle clusters with specific geometric arrangements have been used to demonstrate magnetic activity at optical frequencies^28^,^29^. Therefore, measurement of forces between two magnetic dipoles oscillating at optical frequencies, shown schematically in Fig. 1 (c), could be achieved by measuring the interaction forces between two nanostructures supporting artificial magnetism at a chosen frequency. For example, optical forces between two magnetic dipoles could be measured by measuring the forces between two interacting silicon nano-spheres one placed on a substrate and the other attached to the end of an AFM probe. Experimental work is currently underway to measure the optical forces between two interacting silicon nano-spheres and to map the spatial distributions of incident magnetic fields. We can control the AFM probe over the sample surface using either tapping mode, shear-force feedback or tuning fork in tapping mode to detect different components of the total force 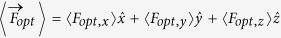 experienced by the AFM tip as shown Fig. 1 (d). For example, the experiments presented in this work were performed only using a tapping mode AFM (with AFM probe tapping along *z* direction) and therefore 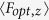 is the only component of the total force that is detected by the AFM cantilever. Also, as shown schematically in Fig. 1 (d), the AFM in shear force feedback mode could be used to detect the *x*-component 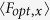 of the total time averaged optical force. Typically, the force detector (like AFM cantilever or tuning fork) supports multiple mechanical resonance harmonics and oscillation modes which have previously been used for multi-frequency force detection and imaging using AFM^5^,^6^,^30^. All experimental results presented in this work recorded the topography information using the first mechanical resonance of cantilever and the optical force signal from the second mechanical resonance of cantilever^5^,^6^.

We chose (a) to map the nanoscale electric field distributions due to tightly focused laser beams using a gold coated AFM probe, (b) to measure the forces between the induced dipole at the apex of a tip and its image dipole in a glass substrate and (c) to measure the forces between the induced dipole at the apex of a tip and another induced dipole in a spherical nanoparticle placed on a glass substrate. In all cases, we assume zero magnetic dipole moment so that both 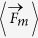
and 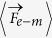
can be neglected.

## Results

**Figure 2:**
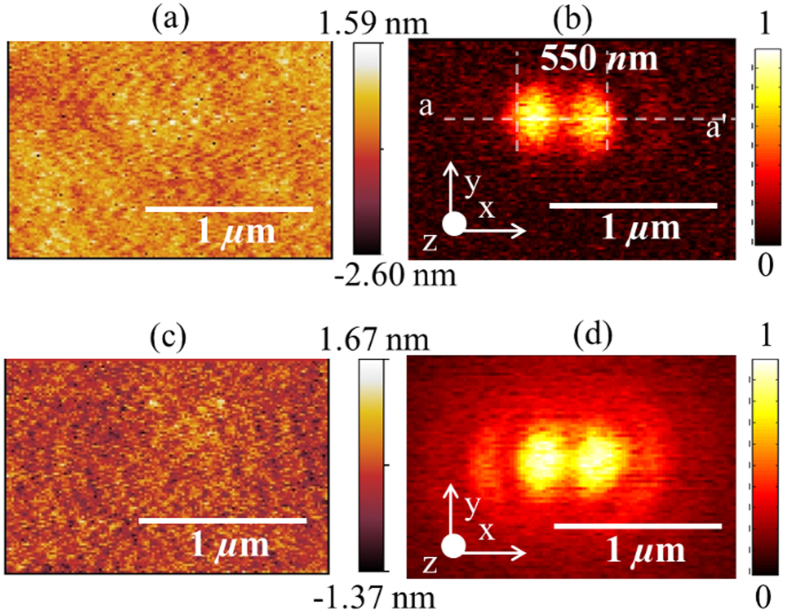
Spatial distributions of (**a**) topography and (**b**) normalized optical force measured experimentally on a clean glass microscope cover slide at 640 nm with light polarized along *x*-axis (**c**) topography of template stripped gold surface (**d**) Normalized optical force measured experimentally on a 10 nm thick template stripped gold surface on a microscope cover slide at 685 nm with light polarized along *x*-axis.

We first present the optical force distribution at the focus of a tightly focused laser beam due to the interaction between a gold coated AFM probe and its image dipole induced in a clean glass substrate of thickness 0.16 mm. The input laser beam (incident on the microscope objective) was polarized along the *x*-axis and the back aperture was over filled to ensure a tight focal spot. The average power of the laser beam was 90 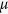
W. The tapping amplitude of the AFM cantilever was 40 nm. The focal spot was raster scanned to simultaneously obtain distributions of topography and optical force. Fig. 2 (a) show the topography and optical force distributions, respectively, recorded at a wavelength of 640 nm. Fig. 2 (c) show the topography and optical force distributions respectively on a 10 nm thick template stripped gold surface^31^ recorded at a wavelength of 685 nm.

**Figure 3:**
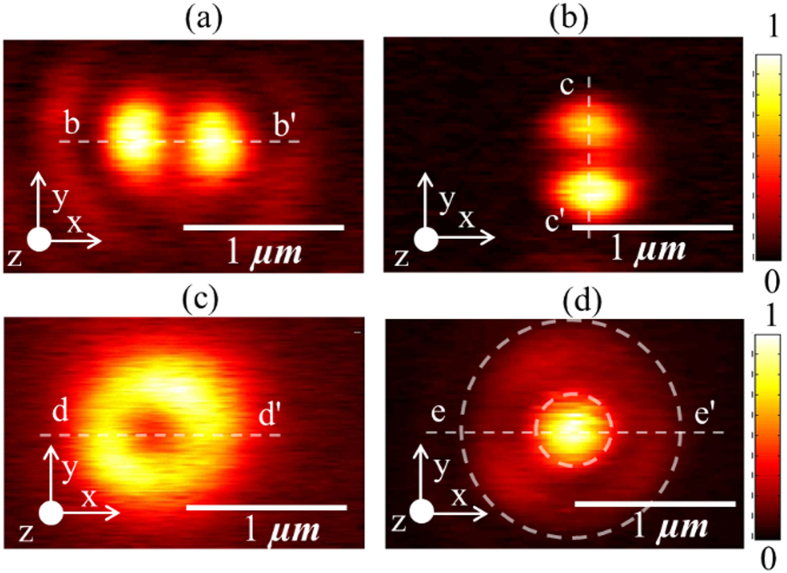
Spatial distributions of (**a**) normalized optical force with polarization along *x* axis (**b**) normalized optical image force with polarization rotated in-plane by 90° when compared to (**a**) (**c**) normalized optical force with azimuthally polarized light (**d**) normalized optical force with radially polarized light.

To further verify our experimental findings, we mapped the focal field distribution at 660 nm for different polarizations of the input (incident on microscope objective) laser beam but using the same gold coated AFM probe for all scans. The average power of the laser beam was 90 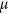
W. Spatial distributions of normalized optical force plotted in Fig. 3 (a) to (d) were obtained for input light polarized along *x* axis, *y* axis (rotated using a half wave plate), azimuthal polarization and radial polarization (obtained using ARCoptix radial polarizer), respectively.

**Figure 4:**
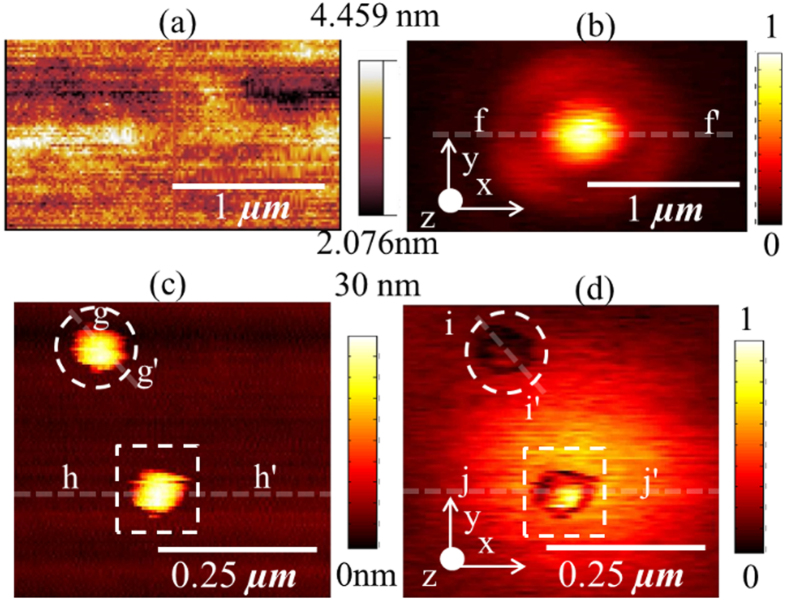
Spatial distributions of (**a**) topography and (**b**) normalized optical image force measured experimentally on clean glass slide at 660 nm with radially polarized input light (**c**) topography and (**d**) normalized optical image force measured experimentally on two gold nanoparticles of radius 15 nm on a cleaned glass surface at 660 nm with radially polarized input light.

In the next series of experiments, we mapped the optical force distribution between a gold coated AFM probe and single gold nanoparticles of radius 15 nm (Sigma-Aldrich 753629) which was drop cast onto a clean glass surface. The nanoparticles were placed in the focal spot of a tightly focused laser beam and the input light was radially polarized at a wavelength of 660 nm. The average power of the laser beam was 90 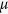
W. The measured optical force distribution of the focal spot (before introduction of the nanoparticles) is plotted in Fig. 4 (b) and corresponds to the plot in Fig. 3 (d). The nanoparticles were then drop cast and raster scanned using a gold coated AFM probe to simultaneously obtain topography and normalized optical force plotted as shown in Fig.4 (c) and (d), respectively. In Fig. 4 (d), we present the optical force distributions of two gold nanoparticles of radius 15 nm illuminated by incident focused light with two different and orthogonal polarizations of light. The first nanoparticle was positioned to be within the central spot with purely longitudinal fields (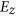) (bound by the dashed box) and the second nanoparticle was located within the circular ring with purely transverse fields (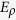) (bound by a dashed circle).

## Discussion

### Mapping the electric field distribution at the focal spot

**Figure 5:**
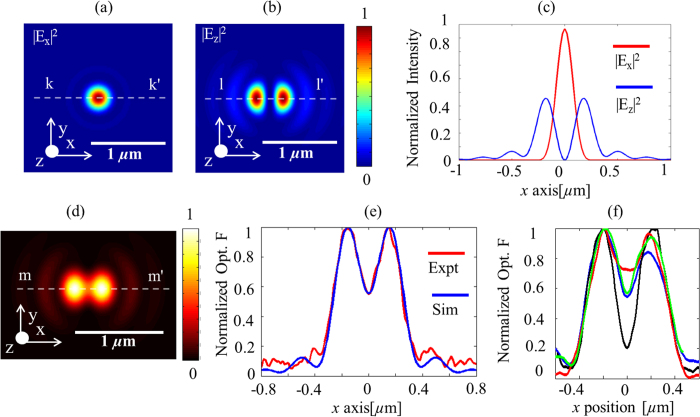
Spatial intensity distributions on glass at 640 nm of (**a**) 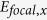 (**b**) 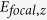 (**c**) comparison of line traces of 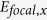
(along k-k’) and 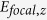 (along l-l’) (**d**) Numerical calculations of normalized optical force obtained by evaluating (1) (**e**) Comparison of line traces of normalized optical force obtained experimentally (line a-a’ in Fig. 2 (b)) and calculations (line m-m’ in (**c**)) showing good agreement.(**f**) Normalized optical force measured experimentally with different gold coated AFM probes.

To map the electric field distribution, we measured the interaction force between the gold coated AFM probe and its image dipole on glass. The measured optical force distribution plotted in Figs. 2 (b), (d) and 3 (a), (b) clearly exhibits two distinct peaks and the focal spot size of 550 nm at 640 nm wavelength in Fig. 2 (b) agrees well with the theoretical prediction of 539 nm. To compare, the optical force was numerically evaluated using the electrostatic approximation,


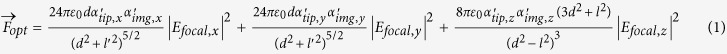


where, 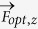, is the *z* component of the total time averaged force in the electrostatic limit, *2l* is the length of the major axis (along the *z*-axis) and *2l*’ is the length of the minor axes, 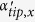, 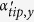 and 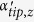 are the real part of electric polarizabilities of the prolate spheroid along the *x*, *y* and *z* axis^27^ and 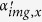, 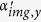 and 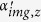 are the real parts of electric polarizabilities of the image dipole along the *x* , *y* and *z* axis. We note that the image dipole polarizabilities are proportional to the polarizabilities of the tip by the scaling factor 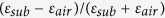
11. From (1), we note that the image force is a sum of three separate contributions from the interaction of the *i*^th^-component of the field with the tip, where *i* = *x*, *y*, *z* and this property could be used to decompose the polarization dependence of the optical field under measurement. The derivation of (1) using dipole-dipole interaction theory in the electrostatic limit is briefly outlined in the Supplementary Information. We calculated the electric field distribution at the focus of a tightly focused laser beam using the plane wave expansion method^11^. We assume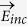 , the electrical field strength of light input to the high *NA* objective lens, to be linearly polarized, for example, along the *x*-axis. The focal field distribution for linearly polarized input light 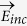
is known to contain components along both the transverse (*x* axis) and axial (*z* axis)^11^ whose numerically computed spatial intensity distributions are shown in Fig. 5 (a), respectively. Figure 5 (c) plots the line traces of the numerically computed focal field intensity distributions for the transverse (line trace *k*-*k*’ from Fig. 5 (a)) and longitudinal (line trace *l*-*l*’ from Fig. 5 (b)) polarizations. Due to the presence of both the *x* and *z* field components in the focal field distribution, 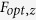
contains force terms due to both the *x* and *z* oriented electric dipoles interacting with the fields. Here, we ignore the *y* component of the focal field distribution as it is much smaller when compared to the *x* and *z* components. To compare with the experimental results plotted in Fig. 2 (b), equation (1) was numerically evaluated at 640 nm. The results plotted in Fig. 5 (d) agreed well with the experimentally measured data plotted in Fig. 2 (b). Furthermore, a line trace extracted from Fig. 2 (b) (along line a-a’) was compared with the trace extracted from Fig. 5(d) (along line b-b’) in Fig. 5 (e). The experimentally measured optical force data agreed well with the numerical calculations demonstrating that measurements of optical forces due to the focal field distributions can be used to estimate the ratio 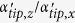 and gain insight into the physical structure of the sharp tip end interacting with the optical field. Experiments were repeated for many different gold coated AFM probes and the results plotted in Fig. 5 (f) indicate a large variability in the repeatability of structural parameters of gold coated AFM probes produced by our gold coating technique which is detailed in the Methods section. Efforts are currently underway to design and fabricate gold coated AFM probes with improved and consistent values of the ratio 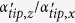 and to characterize the optical properties of such probes. The results in Fig. 5 (f) were all obtained by performing the same experiment used to obtain Fig. 2 (b) but by using different gold coated AFM probes; the line traces were extracted along the curve corresponding to a-a’ in Fig. 2 (b).

**Figure 6:**
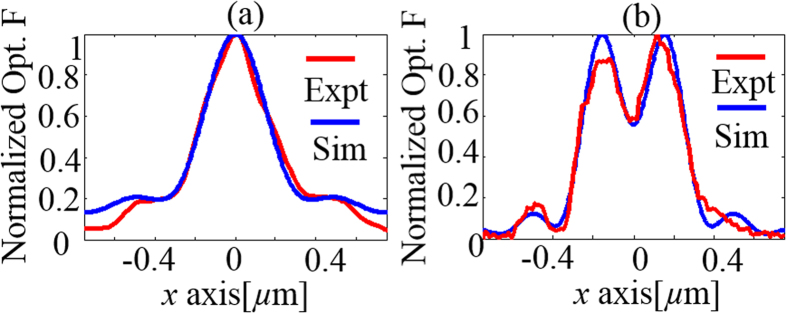
(**a**) Comparison of line traces of normalized optical force obtained experimentally from curve b-b’ in Fig. 3 (a) and numerical calculations obtained by evaluating (1) showing good agreement. (**b**) Comparison of line traces of normalized optical force obtained experimentally from curve e-e’ in Fig. 3 (d) and numerical calculations obtained by evaluating (1) showing good agreement for the same value of ratio used to obtain fit in Fig. 6 (a).

However, since the normalized optical force distributions plotted in Fig. 3 were all performed using the same gold coated AFM probe and at the same wavelength, we expect to fit the experimental data using (1) for the same value of the ratio 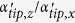 . The line traces extracted from Fig. 3 (a) (along line b-b’) and Fig. 3 (d) (along line e-e’) were compared with an equivalent line traces obtained from numerical calculations in Fig. 6 (a), respectively. Indeed, in both cases, we find the experimentally measured optical force data agrees well with the numerical calculations while maintaining the same value of the ratio 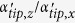 ~16.42 thereby validating the technique proposed in this work. We note that the focal field distribution of a tightly focused laser beam with input radial polarization consists of two distinct regions with orthogonal polarizations. The central peak is a purely longitudinally polarized (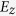) field and the circular ring surrounding the central ring is a purely transverse polarized (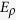) field^32^. In addition, as shown in Fig. 3 (c) (along line d-d’), the Full Width Half Maximum (FWHM) of the dip in the normalized optical force distribution is 337 nm and agrees well with both numerical calculations and previously published data^32^. For the normalized optical force distribution for input radial polarization shown in Fig. 3 (d) (along line e-e’), the FWHM of the focal spot is 356 nm and agrees well with both numerical calculations and previously published data^32^.

To verify that optical force measurements are indeed measuring optically induced forces and not opto-thermal effects, we performed experiments with different samples that have distinctly different thermal diffusion lengths and obtained the normalized optical force distributions shown as Fig. 2 (b),(d). From 33, the thermal diffusion length of glass is 
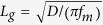
, where *D* is the thermal diffusivity of glass and 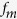
is the optical chopping frequency. The thermal diffusion length of glass is 661 nm at our chopping frequency, whereas our optical force measurement shows a spot size of only 550 nm as shown in Fig. 2 (b). For a 10 nm thick template stripped gold film on glass, the thermal diffusion length is estimated to be ~7.7 *μ*m^33^ at our chopping frequency. However, the measured optical force distribution of the tightly focused laser beam on the template stripped gold surface, shown in Fig. 2 (d), has essentially the same distribution as that of Fig. 2 (b)—a spot size of 550 nm, vastly smaller than the thermal diffusion length clearly indicating the measurement of optically induced forces and not opto-thermal effects.

### Mapping the optical force distribution between a gold coated AFM tip and a single gold nanoparticle

**Figure 7:**
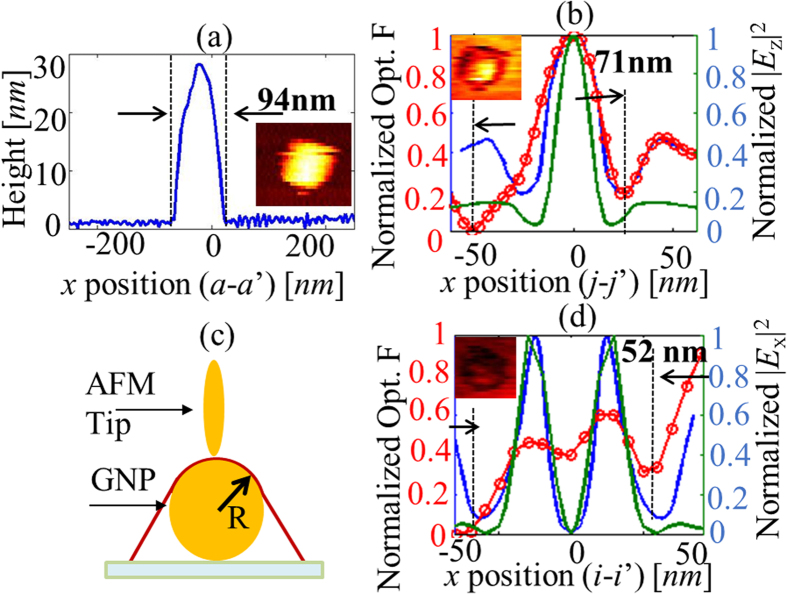
(**a**) line scan of topography indicating feature size of 94 nm (**b**) line scan of normalized optical force (from experiment in red color) with spherical gold nanoparticle in the longitudinal incident field indicating feature size of 71 nm and compared with the line scans of normalized optical force (in blue color) and normalized |*E*_z_|^2^ of only nanoparticle (in green color) from numerical calculations (**c**) Model used in numerical calculations with elliptical gold nanoparticle scanning over the spherical gold nanoparticle and following the theoretical AFM topography curve (**d**) line scan of normalized optical force (from experiment in red color) with spherical gold nanoparticle in the transverse incident field and compared with the line scans of normalized optical force (in blue color) and normalized |*E*_ρ_|^2^ of only nanoparticle (in green color) from numerical calculations. Insets in Fig. 7 (b) and (d) show the zoomed-in images of the measured optical force distributions on the nanoparticles.

The normalized optical force distribution between a gold coated AFM probe and gold nanoparticles of radius 15 nm is shown in Fig. 4 (d). Line traces of the topography and normalized optical forces were extracted from Fig. 4 (c) (along line h-h’) and Fig. 4 (d) (along line j-j’ and i-i’) and plotted in Fig. 7 (a) (in red color) and (d) (in red color), respectively. The inset in Figs. 7 (b) show zoomed-in plots of optical force distributions on the corresponding nanoparticles. The feature width in the line trace of topography plotted in Fig.7 (a) is 94 nm and the broadening is attributed to the convolution of the sample with the probe apex geometry. The width of the measured optical force due to the nanoparticle located in the longitudinal (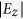) field and extracted from the normalized optical force plotted in Fig. 4 (b) was found to be considerably smaller at 71 nm, or ~*λ*/9.29 at a wavelength of 660 nm. To further study the observed results, we performed numerical calculations using a commercial finite element solver COMSOL Multiphysics. The apex of the gold coated AFM probe was modeled as an ellipsoid composed of gold and the theoretical AFM topography was traced over the gold nanoparticle as shown schematically in Fig 7 (c). The force experienced by the elliptical gold nanoparticle as it traversed the theoretical curve around the gold nanoparticle was calculated by evaluating the Maxwell stress tensor using the electric fields obtained from COMSOL simulations . Line traces of calculated optical forces for both the longitudinal (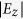) and transverse (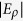) fields using the same theoretical topography curve are also plotted in Fig. 7 (b), respectively (both in blue color) and compared with experimental data. Also, calculations of the EM fields around a single gold nanoparticle of radius 15 nm on glass substrate for the same incident field conditions used to simulate optical force results in Fig. 7 (b) were performed using COMSOL. The values of the |*E*_z_|^2^ and |*E*_ρ_|^2^ components along the theoretical AFM topographical curve were extracted, normalized and plotted along with the normalized force in Figs. 7 (b), respectively (on secondary y axes, in green color). The results of these simple simulations are generally in reasonable agreement with experiment and predict the right trends. The slightly broadened experimental data can be explained by considering the conical shape of the AFM probe.

The measured optical force is in agreement with the calculated field distributions for the bare nanoparticle suggesting that PIFM can be used as a measure of optical field distributions. However, it should be borne in mind that typical of any scanning probe measurement, the optical force maps should be expected to be perturbed by the interaction between tip apex and sample. Indeed, similar to *s*-NSOM, the degree of interaction between tip and sample will determine the perturbation in the measured fields^23^. For the case of strong tip-sample coupling, the normalized optical force maps the interaction forces between the gold coated AFM probe and the sample and indicates the gap mode dominated electric field distribution between the sample and gold coated AFM probe. Such experiments could be very useful to understand the hotspots between closely coupled nanoparticles.

**Figure 8:**
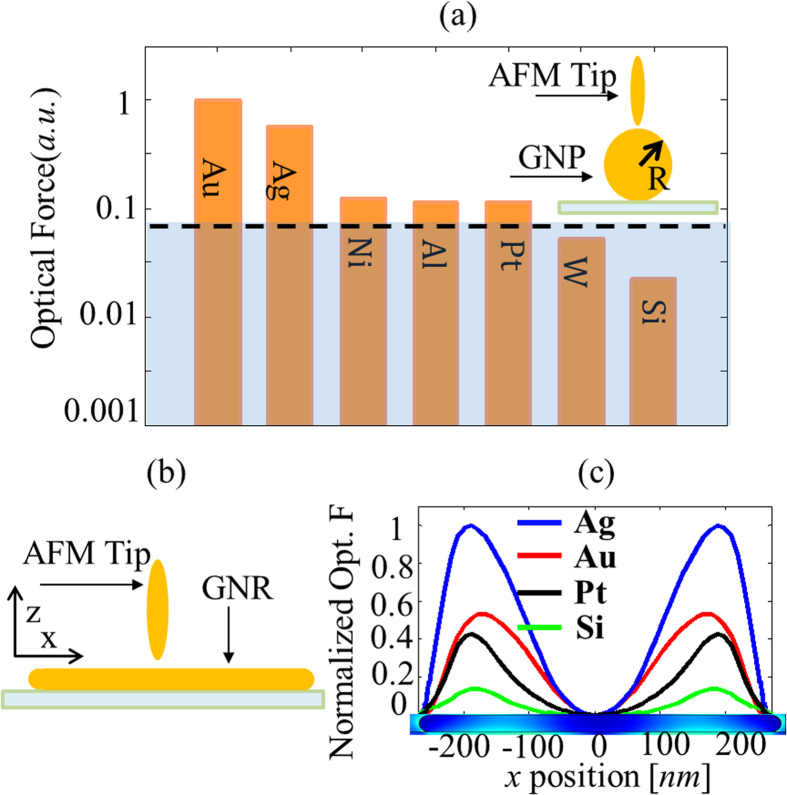
(**a**) Normalized optical force detected by AFM tips coated with different metals while measuring a 30 nm Au particle with incident light at 660 nm in the |E_z_| incident field of different permittivity of the tip calculated by COMSOL (**b**) Model used in numerical calculations with elliptical gold nanoparticle scanning over the 500 nm gold nanorod. (**c**) line scan of normalized optical force of different tip permittivity at 580 nm wavelength on 500 nm gold nano rod in the |Ex| incident field calculated by COMSOL, which shows the experiment setup can measure the Fabry-Perot mode of the gold nano rod.

From our experiments, the peak value of optical force, measured at the second mechanical resonance of the cantilever, between a gold coated AFM probe and the gold nanoparticle is 18.5 times higher than the thermal noise measured at the second mechanical resonance of the cantilever in 8 Hz bandwidth. This peak value of force was obtained from the experiment whose results are plotted in Fig. 4 (d). For this experimental setup, we can identify some AFM probe tip coating materials which could be used to obtain optical force measureable by our experimental setup. The optical force between an AFM probe, (modeled as an ellipsoid with the same parameters as those used to fit our experimental data in Fig. 6), and the gold nanoparticle of radius 15 nm was numerically evaluated using COMSOL for different coating materials. The geometry used in the calculation is shown in the inset of Fig. 8 (a). Plots of the optical force calculated for ellipsoids composed of different materials normalized by the optical force calculated for an ellipsoid of gold. The dashed line indicates the thermal noise measured at the second mechanical resonance of the cantilever and indicates the limit of measurement for our experimental setup. From Fig.8 (a), we see that for the current setup, we can only use gold or silver tips, to obtain measureable optical forces. Other metals such as nickel, platinum and aluminum produce optical forces which are only 2 times above the thermal noise. However, we note that force detection scheme can be vastly improved. By reducing the vibration amplitude of the tip to 1-2 nm, bringing the tip much closer (within 1-2 nm) to the sample and placing the experiment setup in a rough vacuum (thereby increasing the Q of the cantilever 100 times), and lowering the temperature from 300 K to 3 K, the minimum detectable force and force gradient can be reduced by several orders of magnitude down to the attonewton range.

To demonstrate theoretically that we can use our technique to map modal distributions, we used COMSOL simulations to calculate the optical force on an ellipsoidal AFM tip scanning a 500 nm gold nano rod driven by an optical field at 580 nm polarized along its *x*- axis as shown in Fig. 8 (b). The calculated optical force map for an AFM tip scanning over the nano rod clearly reveals the Fabry-Perot modes of the nano rod. As expected, changing the permittivity of the AFM tip changes the contrast of the optical force signal shown in Fig.8 (c).

In conclusion, we experimentally demonstrated the application of Atomic Force Microscopy (AFM) based Photo Induced Force Microscopy (PIFM) to map optical near-fields. The spatial resolution is limited only by the AFM probe geometry. We mapped the spatial electric field distributions of tightly focused laser beams with linear, radial and azimuthal polarizations by measuring the optical force between a gold coated AFM probe and its image dipole on a glass substrate. We derived a dipole-dipole theory to model the image force and showed that the experimentally measured data agreed well with our theoretical predictions. We propose that PIFM can be used to characterize optical properties of nanoprobes. Finally, we mapped the nanoscale optical interaction force between a gold coated AFM probe and a spherical gold nanoparticle of radius 15 nm. PIFM allows for background free, thermal noise limited mechanical imaging of optical phenomenon over a wide wavelength range from visible to RF with detection sensitivity limited only by AFM performance.

## Methods

### Experimental setup

**Figure 9:**
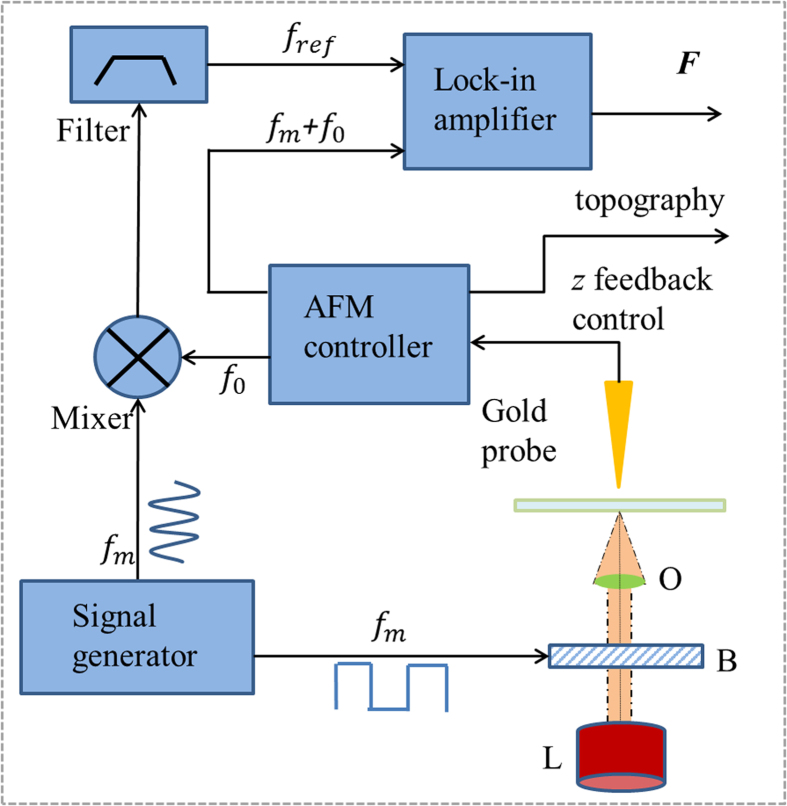
Schematic of Photo Induced Force Microscopy (PIFM) used to measure the optically induced force ***F*** by locking onto the modulated optical field, generated by a laser (L). Incident light is modulated at frequency *f*_m_ by a Bragg cell (B) and focused on to the sample by oil immersion objective lens (O). The cantilever or tuning fork detects this modulated field on one of its resonant frequencies while the electronic reference is generated by mixing the modulation frequency (*f*_m_) and the detector self-oscillation frequency (*f*_0_) in a mixer and subsequent band-pass filtering.

The experimental setup was built around a commercial AFM (Veeco Caliber) operating in the tapping mode and the schematic of the experimental setup is shown in Fig. 9. The optical field under measurement, generated by a laser of suitable wavelength, was modulated using a Bragg cell modulator at the modulation frequency *f*_m_ and focused on the sample by using an oil immersion objective with *NA* = 1.45. The incident optical field induces a dipole at the end of the AFM probe when illuminated by a tightly focused laser beam modulated at frequency *f*_m_. The attractive gradient optical force (***F***) between the induced dipole on the tip and its image dipole in the substrate is also modulated at frequency *f*_m_ which in turn modulates the AFM cantilever at its first mechanical resonance frequency *f*_0_ generating sidebands at *f*_0 _+ *f*_m_ and *f*_0_ − *f*_m_. We locate the sideband *f*_0_ + *f*_m_ on top of the second mechanical resonance frequency of the AFM cantilever thereby utilizing the high *Q* of the second resonance of the AFM cantilever^30^ to enhance optical force signal. The optical image force signal is then detected using a lock-in amplifier. An electronic reference is derived by mixing the modulating frequency *f*_m_ with the cantilever resonance frequency *f*_0_ in a double balanced mixer followed by a bandpass filter centered at *f*_0 _+ *f*_m_. In our experiments, we chose *f*_m_ ~ 360 kHz and the sideband frequency *f*_0 _+ *f*_m_ ~ 425 kHz. All experiments were performed by focusing light using an oil immersion objective (PlanApo 100x) with *NA* = 1.45 on a 0.16 mm thick clean glass microscope cover slide.

### Tip preparation

Gold coated AFM cantilever probes were prepared by sputter coating (South Bay technology) commercial bare Silicon AFM cantilever probes (AppNano Forta **k** = 1.6 N/m, *f*_0_ ~ 65 kHz) with 25 nm gold on a 2 nm chromium adhesion layer.

### Template stripped gold

A piece of solvent clean silicon is sputter coated (south bay technology) with 10 nm of gold. Optical UV curable epoxy (Thorlabs NOA 61) is used to glue the glass surface to the gold which is on the silicon. Once the silicon is stripped, the ultraflat gold surface on the glass cover slide has a roughness of 0.15 nm–0.35 nm.

### Nano particle sample preparation

Cleaned glass slides were prepared by rinsing 1.6 mm thick glass cover slides in acetone, methanol and isopropanol respectively. Gold nanoparticle of radius 15 nm (Sigma-Aldrich753629) were prepared by centrifuging the commercial aqueous nanoparticle in DI water at 13,000 rpm for 20 minutes to remove surfactants. Then, 10 *μ*l of the resulting nanoparticle solution with particle concentration 2 × 10^11^/ml was drop cast onto a clean glass surface. After one hour, the sample was gently blow dried.

### Numerical calculations

The numerical data was fitted to the experimental data shown in Fig. 5 (e) using the ratio 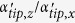
~16.91 which was obtained using the following parameters: *l* = 7.5 nm, *l*’=1.97 nm, *d* = *2l* nm (assume AFM probe in contact with glass surface) and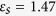. The numerical data was fitted to the experimentally measured data shown in Figs. 6 (a),(b) using the ratio 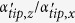 ~16.42 which was obtained using the following parameters: *l* = 7.5 nm, *l*’=1.898 nm, *d* = *2l* nm (assume AFM probe in contact with glass surface) and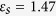. The optical constants of gold and silicon used in all calculations in the work were obtained from^34^ and^35^, respectively. The optical constants of other metals used to obtain results plotted in Fig. 8 (a) were obtained from^36^. In Fig. 7 (c), the gold coated tip modeled as an ellipsoid has *l* = 40 nm, *l*’=4 nm.

## Additional Information

**How to cite this article**: Huang, F. *et al*. Imaging Nanoscale Electromagnetic Near-Field Distributions Using Optical Forces. *Sci. Rep.* **5**, 10610; doi: 10.1038/srep10610 (2015).

## Supplementary Material

Supplementary Information

## Figures and Tables

**Figure 1:**
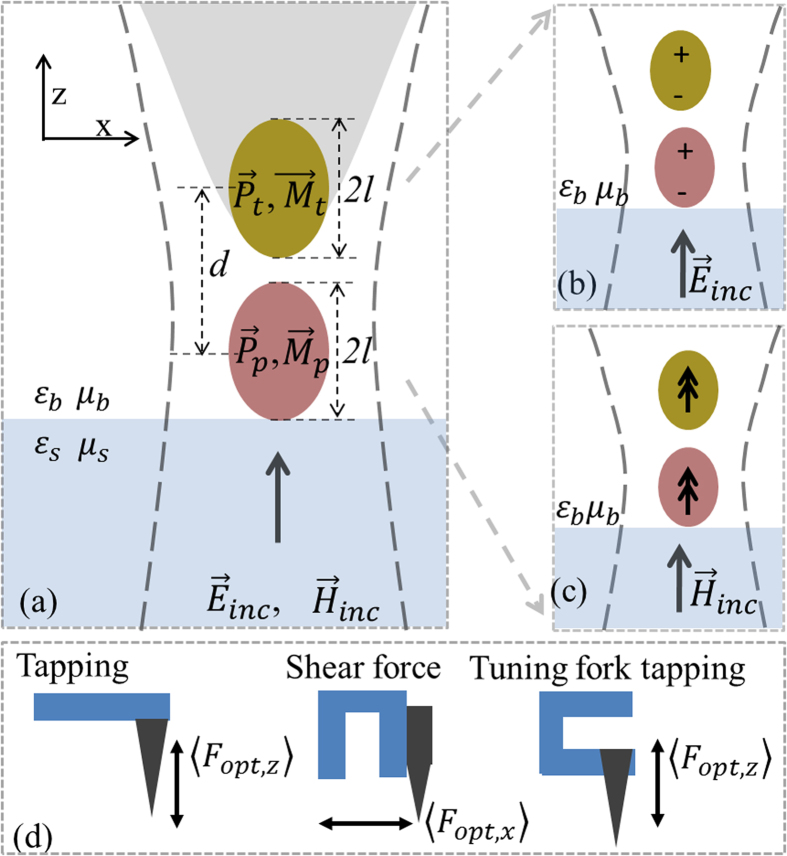
(**a**) Schematic of the tip-sample interaction under illumination by tightly focused laser beam with the AFM probe tip modeled as a sub-wavelength magneto-dielectric particle and the tip-sample interaction modeled as (**b**) electric and (**c**) magnetic dipole-dipole interactions (**d**) Three different AFM working modes include the tapping mode AFM which detects force in *z* direction, tuning fork AFM working in shear force mode detects force in *x* direction while tuning fork AFM working in tapping mode detects force in *z* direction.
