# Supplementary material for: Imaging Nanoscale Electromagnetic Near-Field Distributions Using Optical Forces
Source: Sci Rep. 2015 Jun 15;5:10610. doi: 10.1038/srep10610 (PMC5155520; doi:10.1038/srep10610)
Supplement: Supplementary Information [file srep10610-s1.pdf]

# Supplementary Information

## Imaging Nanoscale Electromagnetic Near-Field Distributions Using Optical Forces


*Fei Huang[1], Venkata Ananth Tamma[2], Zahra Mardy[1], Jonathan Burdett[2] and H. Kumar Wickramasinghe [1*]*

*[1] Department of Electrical Engineering and Computer Science, 142 Engineering Tower, University of California, Irvine, USA*

*[2] CaSTL Center, Department of Chemistry, University of California, Irvine, USA*


### Computation of electric dipole-dipole interaction force

The total force experienced by the AFM probe tip is $\vec{F}_{tot} = \vec{F}_{int} + \langle \vec{F}_{opt} \rangle$, where, $\vec{F}_{int}$ is the total tip-sample interaction forces consisting of all van der Waals forces, meniscus forces, chemical and Casimir forces, and $\langle \vec{F}_{opt} \rangle$ is the time-averaged optical force on the AFM probe tip due to its interaction with the incident field and particle dipole. Due to the presence of both electric and magnetic dipoles, $\langle \vec{F}_{opt} \rangle$ can be written as the sum, $\langle \vec{F}_{opt} \rangle = \langle \vec{F}_{e} \rangle + \langle \vec{F}_{m} \rangle + \langle \vec{F}_{e-m} \rangle$ ,where, $\langle \vec{F}_{e} \rangle$ is the time-averaged force experienced due to the electric dipole with spatially non-varying moment $\vec{P}_t$ , $\langle \vec{F}_{m} \rangle$ is the time-averaged force experienced due magnetic dipole with spatially non-varying moment $\vec{M}_t$ , $\langle \vec{F}_{e-m} \rangle$ is the interaction force due to coupling between the electric and magnetic dipoles. Here, $\vec{E}_{t,loc}$ and $\vec{B}_{t,loc}$ are the local electric and magnetic fields experienced by the AFM probe tip respectively and are given by the sum of the incident field and the fields scattered by the particle.

Since we chose to map the nanoscale electric field distributions, we coat the AFM probe with 30 nm thick layer of gold. Since the sample does not have magnetic response at optical frequency, the force due to magnetic dipole-dipole interaction is neglected. The gold coated AFM probe is modeled as an ellipsoidal dielectric nanoparticle. The total time average electric force [S1] is

$$\langle \vec{F}_{e} \rangle = \frac{1}{2} \Re \left\{ \vec{P}_t \left( \nabla \otimes \vec{E}_{t,loc} \right) \right\}, \tag{1}$$

where, $\vec{E}_{t,loc}$ is the total electric field experienced by the gold coated AFM probe and is given by the sum of the incident field and the fields scattered by the particle dipole [S2]. Equation (1) can also be written as

$$\langle \vec{F}_{e} \rangle = \frac{1}{4} \alpha' \nabla \left| \vec{E}_{t,loc} \right|^2 + \frac{k}{2n} \alpha'' \Re \left( \vec{E}_{t,loc} \times \vec{B}^{*}_{t,loc} \right) + \frac{1}{2} \alpha'' \Im \left( \left( \vec{E}^{*}_{t,loc} \bullet \nabla \right) \vec{E}_{t,loc} \right) \tag{2}$$

where, the electric polarizability of the dielectric nanoparticle $\alpha = \alpha' + i\alpha''$ with real and imaginary parts, $\alpha'$ and $\alpha''$ , respectively. In (2), we associate the first term with the electric dipole-dipole interaction force, the second term with the scattering force, and the third term with a curl force associated to the non-uniform distribution of the spin density of the electric field. We note that the scattering force along the *z*-direction is much smaller than the gradient force [S2], [S3]. Also since the scattering force is substantially constant in the vicinity of the focal spot and the typical vibration amplitude of the AFM cantilever is only 40 nm, we are not able to detect this force in our experiments. Furthermore, the curl force was zero due to the uniform distribution of the spin density of the electric field in the focal plane.

Experiments were performed using a tapping mode AFM and therefore only $\langle F_{opt,z} \rangle$ is the only component of the total force that is detected by the AFM cantilever. $\langle F_{opt,z} \rangle$ can be expanded in terms of the fields as

$$\langle F_{opt,z} \rangle = \Re\left( P_{tip,x}\frac{\partial E_{tip,z}}{\partial x} + P_{tip,y}\frac{\partial E_{tip,z}}{\partial y} + P_{tip,z}\frac{\partial E_{tip,z}}{\partial z} \right) \quad (3)$$

where, $p_{tip,x}$, $p_{tip,y}$ and $p_{tip,z}$ are the components of the electric dipole moment along the *x*, *y* and *z* axis respectively and $E_{tip,z}$ is *z* components of the total electric field on the AFM probe tip. We model the interacting region of the tip as a prolate spheroid with $l > l'$ , where, $2l$ is the length of the major axis (along the *z*-axis) and $2l'$ is the length of the minor axes [S4]. We note that $P_{tip,x} = P_{tip,y}$ assuming symmetry of the AFM tip. The configurations of the dipole-dipole interaction model for $E_z$ and $E_x$ incident fields of the tip dipole and the image dipole are shown in Figs. S1 (a) and (b) respectively.

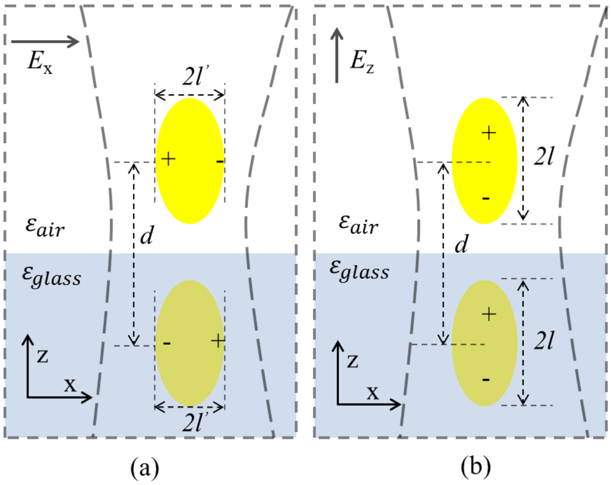


Fig. S1: Tip sample interaction due to electric field modeled as the dipole-dipole interaction along (a) *x* direction, (b) *z* direction.

Assuming $l, l' << \lambda$ and using the electrostatic approximation [S2], we obtain

$$\Re\left( P_{tip,x}\frac{\partial E_{tip,z}}{\partial x} \right) \approx \frac{24\pi\varepsilon_0 d\alpha'_{tip,x}\alpha'_{img,x}}{\left(d^2 + l'^2\right)^{5/2}} \left|E_{focal,x}\right|^2, \quad (4)$$

$$\Re\left( P_{tip,y}\frac{\partial E_{tip,z}}{\partial y} \right) \approx \frac{24\pi\varepsilon_0 d\alpha'_{tip,y}\alpha'_{img,y}}{\left(d^2 + l'^2\right)^{5/2}} \left|E_{focal,y}\right|^2, \quad (5)$$

$$\Re\left( P_{tip,z}\frac{\partial E_{tip,z}}{\partial z} \right) \approx \frac{8\pi\varepsilon_0 \alpha'_{tip,z}\alpha'_{img,z}(3d^2 + l^2)}{\left(d^2 - l'^2\right)^{3}} \left|E_{focal,z}\right|^2, \quad (6)$$

where, $\alpha'_{tip,x}$ , $\alpha'_{tip,y}$ and $\alpha'_{tip,z}$ are the real part of electric polarizabilities of the prolate spheroid along the $x, y$ and $z$ axis and $\alpha'_{img,x}$ , $\alpha'_{img,y}$ and $\alpha'_{img,z}$ are the real part of electric polarizabilities of the image dipole along the $x$ , $y$ and $z$ axis. We note that the image dipole polarizabilities are proportional to the polarizabilities of the tip by the scaling factor $(\varepsilon_{sub} - \varepsilon_{air})/(\varepsilon_{sub} + \varepsilon_{air})$ [S2]. In deriving (4)-(6), we have neglected terms with fast spatial decay rates of $d^{-7}$ as our experimental setup is unable to detect signals with such rapid decay. Finally, the total detectable optical image force experienced by the tip is given by the sum of (4)-(6)

$$\vec{F}_{opt} = \frac{24\pi\varepsilon_0 d\alpha'_{tip,x}\alpha'_{img,x}}{\left(d^2 + l'^2\right)^{5/2}}\left|E_{focal,x}\right|^2 + \frac{24\pi\varepsilon_0 d\alpha'_{tip,y}\alpha'_{img,y}}{\left(d^2 + l'^2\right)^{5/2}}\left|E_{focal,y}\right|^2 + \frac{8\pi\varepsilon_0\alpha'_{tip,z}\alpha'_{img,z}(3d^2 + l^2)}{\left(d^2 - l'^2\right)^3}\left|E_{focal,z}\right|^2 . \quad (7)$$

**References**


S1. Nieto-Vesperinas, M., Sáenz, J. J., Gómez-Medina, R. & Chantada, L. Optical forces on small magnetodielectric particles. *Opt. Express.* ***18***, 11428-11443 (2010).

S2. Novotny, L. & Hecht, B. *Principles of Nano-Optics*. (Cambridge Univ. Press, Cambridge, UK, 2006).

S3. Kawata, S., Ohtsu, M., Irie, M.Near-Field Optics and Surface Plasmon Polaritons (*Springer Science & Business Media*, 2001)

S4. Bohren, C. F., Huffman, D. R. Absorption and Scattering of Light by Small Particles. (*Wiley Interscience*, New York, 1983)
